# Classification of subdural hematomas: proposal for a new system improving the ICD Coding Tools

**DOI:** 10.3389/fneur.2023.1244006

**Published:** 2023-10-11

**Authors:** Anne-Marie Langlois, Charles J. Touchette, David Mathieu, Christian Iorio-Morin

**Affiliations:** Division of Neurosurgery, Department of Surgery, Université de Sherbrooke, Sherbrooke, QC, Canada

**Keywords:** subdural hematoma, trauma, intracranial hemorrhage, ICD, chronic subdural hematoma

## Abstract

**Background:**

The International Statistical Classification of Diseases (ICD) classifies subdural hematoma (SDH) as traumatic or non-traumatic. In clinical settings, however, SDH is typically described as either acute or chronic.

**Objective:**

The goal of this study was to assess how the ICD Coding Tools captures the clinical terminology and propose an improved classification that would increase the system’s usefulness in administrative, statistical and research applications.

**Methods:**

We performed a retrospective analysis of patients who presented to our center with an ICD diagnostic code for either traumatic or non-traumatic SDH. A qualitative analysis of patients’ charts was performed to identify elements relevant to management and prognosis, following which a meeting between expert investigators was held to elaborate a new classification of SDH. Imaging from all patients was then reviewed and cases were reclassified according to our proposed system.

**Results:**

A total of 277 SDH cases were included. Themes documented in the charts included chronicity, etiology, side, and symptoms. We created a new classification which distinguishes acute SDH (aSDH) from membrane-associated SDH (mSDH). aSDH were further divided into traumatic aSDH (taSDH) and non-traumatic aSDH (ntaSDH), while mSDH were divided into acute on chronic (a/cSDH), subacute (sSDH) and chronic (cSDH) categories.

**Conclusion:**

The ICD coding system correctly identifies taSDH and ntaSDH. However, it remains non-specific for mSDH. We propose this new SDH classification system to better capture chronicity and etiology – factors felt to impact management and prognosis.

## Introduction

Subdural hematoma (SDH) is among the most frequent reasons for neurosurgical consult (1, 2). Acute SDH (aSDH) typically results from the traumatic laceration of a bridging vein, leading to a subdural accumulation of blood (3). Large aSDH are a neurosurgical emergency and require prompt evacuation through a craniotomy (4, 5), with or without bone flap removal (6). Small, asymptomatic aSDH, on the other hand, are usually observed. While most will resolve spontaneously, a proportion of aSDH may progress into chronic SDH (cSDH) (3, 7). cSDH have a completely different pathophysiology and prognosis. They usually result from repeated bleeding from the SDH outer membrane, which results from an inflammatory reaction to the initial aSDH. Anticoagulant and fibrinolytic factors within the hematoma promote these recurrent bleeds, preventing membrane thrombosis and driving cSDH growth. Acute rebleeding within the cSDH can occur spontaneously and patients usually present with non-specific symptoms of imbalance, headaches, confusion with or without focal neurologic deficits. Surgical evacuation is the mainstay of treatment of cSDH, and typically consists of burr-hole evacuation (3).

The International Statistical Classification of Diseases and Related Health Problems is a diagnostic tool that allows for the standardized reporting and classification of all diseases. As a WHO-sponsored effort, it is the most widely used framework for reporting medical diagnoses for administrative, financial, statistical, and research purposes. In its latest revision (ICD-11), SDH is classified as either *traumatic* (code NA07.6) or *nontraumatic* (code 8B02). The origin and rationale for this mechanistic distinction is unclear (2, 3, 8).

When designing the Tranexamic Acid in Chronic Subdural hematomas (TRACS) study (9), we queried our hospital database for cSDH incidence and were unable to reliably distinguish aSDH from cSDH using the ICD diagnostic codes, which were in their 10th version at the time yet remained identical to the current ICD-11 categories. This same challenge was reported in 2016 by a group from Denmark (8). Among the 936 cases of SDH they reviewed, cSDH represented 57% of overall cases, of which 56% had been recorded under code S06.5 and 54% under code I62.0 using the previous ICD-10 diagnostic codes. They concluded that the ICD classification did not allow for proper distinction between cSDH and aSDH, as cSDH were practically equally classified under both *traumatic* and *nontraumatic* codifications (8). In January 2022, the 11th version of the ICD Coding Tools, the ICD-11, was adopted, however the classification of SDH remains unchanged apart from its attributed diagnostic codes, i.e., *traumatic* (code NA07.6, previously S06.5) or *nontraumatic* (code 8B02, previously I62.0) SDH.

Because the pathophysiology, management and prognosis of SDH depend not on the mechanism of the injury as much as on the existence of a chronic outer membrane, we propose a new classification of SDH that would better capture this element, with the goal of improving the classification’s usefulness in administrative, statistical and research applications.

## Methods

We reviewed all cases of SDH seen at our center between 2016 and 2017, at which time the ICD-10 codes were in use. Patients were included in the study if they had been assigned the ICD-10 diagnostic codes for either *traumatic* (S06.5) or *nontraumatic* SDH (I62.0) upon admission or throughout their hospital stay. The list of all patients included was then crossed with the TRACS screening log to validate that no cases were missed by the medical archivists. A qualitative analysis of the diagnoses written on the consult was performed to identify elements deemed relevant to management and prognosis by the treating physicians. After the cases were reviewed, a meeting was held between the investigators to elaborate a classification that would capture the diagnoses used in the patient charts. Then, imaging from all patients was reviewed and cases were reclassified according to our proposed system.

This study was approved by our Research Ethics Board (*Comité d’éthique de le recherche du CIUSSS de l’Estrie-CHUS*, FWA 00005894 and IRB00003849) under the protocol 14-213. Because of the retrospective and administrative nature of the study, consent from patients was not required.

## Results

### Elements relevant to SDH management

**Figure 1:**
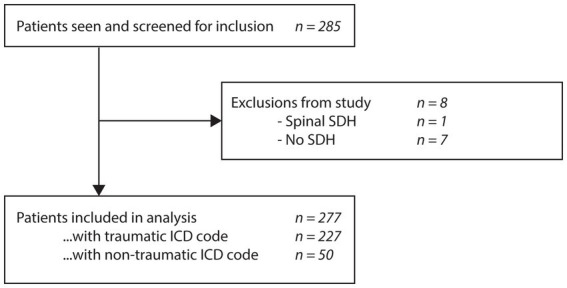
Flow diagram of patient selection.

A total of 285 patients were screened and 277 met the inclusion criteria for analysis (Figure 1). Upon chart review, themes most commonly documented in the diagnostic summary of the initial consultation were:

- Acute vs. subacute vs. chronic vs. acute on chronic vs. subacute on chronic vs. hygroma.
- Right-sided vs. left-sided vs. bilateral.
- Symptomatic vs. asymptomatic.
- Primary vs. recurrent vs. secondary (ex: in the setting of TBI, aneurysmal SAH or ICH).

Typical diagnoses included “right aSDH secondary to severe TBI,” “right, asymptomatic cSDH,” “symptomatic recurrence of left cSDH” or “bilateral, symptomatic, acute on chronic SDH.” The concept of traumatic vs. non-traumatic was not specified in the final diagnosis in most cases, even though a trauma might have been described in the history of present illness. Trauma was mostly documented in cases of aSDH.

### Proposed classification

In light of the elements identified above, we propose a new classification detailed in Table 1. The system starts by assessing the chronicity of the lesion, which appeared as the most important factor in determining management. New, acute lesions are classified as aSDH whereas we suggest the name *membrane-associated SDH* (mSDH) to refer to SDH resulting from the bleeding of a membrane (the entity considered as the classical chronic SDH). Etiology is then described for aSDH, while imaging aspect is used to further detail mSDH. The presence of symptoms and the side of the lesion were not felt to add much to the system and were thus not used in the classification.

**Table 1:** Proposed classification for SDH.

**I. Acute Subdural Hematoma (aSDH)** i. Traumatic (taSDH) ii. Non-traumatic (ntaSDH)
**II. Membrane-associated subdural hematoma (mSDH)** i. Acute on chronic (a/cSDH) ii. Subacute (sSDH) iii. Purely chronic (cSDH)

The *aSDH* entity is defined as a hyperdense subdural collection that is assumed to be new (6). We divided this entity with the *traumatic* (taSDH) and *non-traumatic* (ntaSDH) subcategories since we believe the mechanism of injury between those two categories is very different and might have prognostic implication within the aSDH category (6, 10). *Traumatic aSDH* therefore encompasses all aSDH resulting from an immediate trauma and this code can be viewed as a form of SDH-complicated TBI. *Non-traumatic aSDH* captures cases secondary to processes other than TBI, such as spontaneous ICH or aneurysmal SAH contaminating the subdural space. The radiological evolution of the aSDH (resolution versus conversion to mSDH) is expected to be similar with both *traumatic* and *non-traumatic* aSDH, while the patient’s prognosis in these cases will likely depend on the mechanism (i.e., severe TBI versus ICH versus SAH).

**Figure 2:**
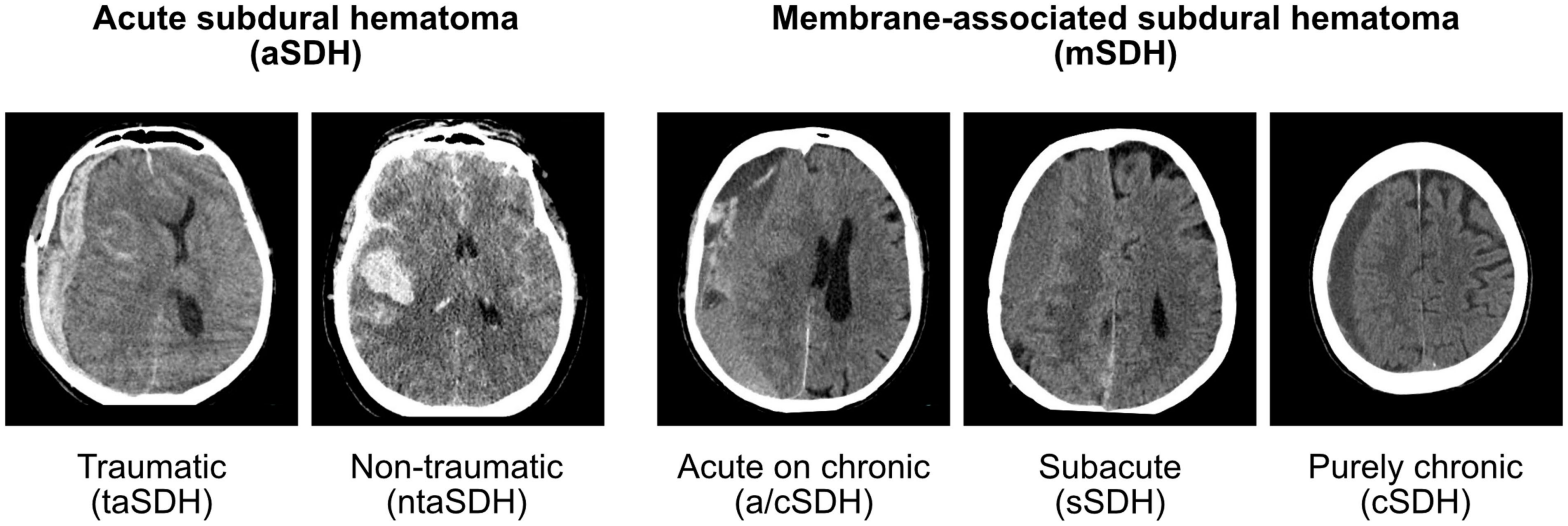
Representative cases for each diagnosis within our new classification.

On the other hand, *mSDH* refers to a known or assumed *aSDH* that, instead of resolving, went on to develop an organized outer membrane which is now the main driver of the pathology (3). The prognosis of *mSDH* is probably independent of the initial aSDH and rather depends on the vascularity of the membrane, the occurrence of rebleeding and patient factors such as age (1–3, 11, 12), comorbidities (7, 11, 12), and the need for antithrombotic and anticoagulant medications (3, 13, 14). As will be discussed below, a traumatic incident can sometimes be identified, although many patients might have suffered only a minor fall or hit that was never documented. In our opinion, further classifying mSDH into traumatic or non-traumatic subcategories would be unreliable and irrelevant as it would not inform management or prognosis. In our qualitative chart review, mSDH were usually further described as *acute on chronic* (a/cSDH), *subacute* (sSDH), or *purely chronic* (cSDH). SDH were classified as *acute on chronic* if there were hyperdense components within the hypodense subdural collection; as *subacute* if the collection was isodense in relation to the brain parenchyma; and finally as *purely chronic* when the collection was homogeneously hypodense in relation to the brain parenchyma. While we have no evidence that this subclassification has any prognostic implication, it could relate to the mode of presentation and is typically documented in CT scan reports. As such, we decided to include these categories in our proposed classification as it allows the continuing use of current clinical terminology. Figure 2 presents representative examples of lesions from each category.

Finally, some chronic subdural collections identified in the charts were proven to be hygromas. Hygroma is defined as a subdural collection of CSF (3, 15, 16). Hygroma does not exist in the current ICD classification, yet is a well described entity with a completely benign course and usually no need for surgical intervention (3). Because it is part of the differential diagnosis of mSDH, we believe it would be relevant to add it to a future ICD classification as a separate entity, although it is not formally part of our proposed classification.

### Comparison between ICD-10 and our proposed classification

Among the 285 patients in our cohort, 229 (80%) were initially classified as *traumatic SDH* (code S06.5) and 56 (20%) as *non-traumatic SDH* (code I62.0) as per the ICD-10 classification. In Table 2, we present a cross comparison of the ICD classification and our proposed system.

**Table 2:** Distribution of SDH cases under ICD-10 and our proposed classification.

ICD-10	Acute SDH		Membrane-associated SDH				Hygromas	TOTAL
	Traumatic	Non-traumatic	Total	Acute on chronic	Sub-acute	Purely chronic		
Traumatic	132 (97.8%)	4 (28.6%)	86 (70.5%)	39 (72.2%)	33 (80.5%)	14 (51.9%)	5 (83.3%)	227 (82%)
Non-traumatic	3 (2.2%)	10 (71.4%)	36 (29.5%)	15 (27.8%)	8 (19.5%)	13 (48.1%)	1 (16.7%)	50 (18%)
TOTAL	135 (100%)	14 (100%)	122 (100%)	54 (100%)	41 (100%)	27 (100%)	6 (100%)	277 (100%)

*Note that the 1 case of spinal SDH and the 7 cases of no SDH were not included in the table above.

Among the SDH initially classified as *traumatic* (*n* = 229) according to ICD-10 codes, 57.6% were reclassified as *taSDH*, 2% as *ntaSDH*, and 37.6% as *mSDH*. In contrast, patients admitted under the *non-traumatic SDH* code (*n* = 56) were reclassified as *taSDH* in 5.4% of cases, as *ntaSDH* in 17.9% of cases and as *mSDH* in 64.2% of cases. Upon patient chart and imaging review of all SDH recorded under either *traumatic* or *non-traumatic* codes, 6 were reclassified as *hygromas*, 1 as a *spinal* SDH and 7 as *no* SDH. It is interesting to note that the case of spinal SDH resulted from a spinal dural fistula, which developed in the context of a traumatic spinal subarachnoid hemorrhage. While the pathogenesis of spinal SDH is unknown, it is indeed proposed that it can often result from a spinal SAH caused by trauma or any increase in intra-abdominal or intra-thoracic pressure such as was the case here (17). However, considering it remains a rare entity which does not enter the differential diagnosis of *mSDH*, we opted to simply exclude this entity from our classification.

When starting from our proposed classification and analyzing the official admission code, 97.8% of all *taSDH* (*n* = 135) had accordingly been recorded under *traumatic* upon admission, while 71.4% of *ntaSDH* (*n* = 14) were coded under *non-traumatic* ICD codes. Regarding the 122 cases of *mSDH* identified upon review (*n* = 122), 70.5% had been initially classified as *traumatic* and 29.5% as *non-traumatic*. Interestingly, among these cases of *mSDH*, 44.3% were subclassified as a/cSDH, 33.6% as sSDH and 22.1% as cSDH. Table 3 presents the sensitivity, specificity, positive predictive value and negative predictive value of the *traumatic* ICD code to detect *taSDH* and the *non-traumatic* ICD code to detect mSDH.

**Table 3:** Performance of ICD-10 codes to detect taSDH and mSDH.

	Using ICD-10 *traumatic* code to detect *taSDH*	Using ICD-10 *traumatic* code to detect *mSDH*	Using ICD-10 *non-traumatic* to detect *mSDH*
Sensitivity	98%	70%	30%
Specificity	30%	2%	79%
Positive predictive value	61%	39%	92%
Negative predictive value	92%	8%	61%

## Discussion

Membrane-associated subdural hematoma remains a common disease which typically occurs in older patients often resulting from less severe injury than acute subdural hematoma. Both entities vary greatly in their clinical presentation, preferred management approach and overall prognosis. In this study, we showed that chronicity, lesion side, presence of symptoms and etiology are the most reliably documented factors in the charts of SDH patients. We postulated that these data inform management more than the concept of trauma and designed a classification that would capture this information.

When comparing our patients’ diagnosis between our classification and the previous ICD-10 diagnostic codes, it is notable that the ICD coding remained adequate for classifying *taSDH* (97.8% sensitivity) and to a certain extent *ntaSDH* (71.4% sensitivity). Nonetheless, it remains inaccurate in classifying *mSDH* (29.5% sensitivity) and its subcategories, *acute on chronic*, *subacute* and *purely chronic*. In fact, all patients initially coded as *traumatic* by the ICD codes were later almost equally reclassified as either *taSDH* and *mSDH* according to our classification. These findings suggest that ICD is unable to adequately differentiate acute from membrane-associated SDH – a conclusion also reached by the Danish team in 2016 (8). And while the ICD Coding Tool appears sensitive to identify *taSDH* and *ntaSDH*, the categories are contaminated by *mSDH*, which are distributed between the *traumatic* and *non-traumatic* categories, making this classification non-specific (Table 3). This remains true for the newer ICD-11 Coding Tools, which encompasses an identical classification of SDH with simply updated diagnostic codes.

Between 2013 and 2017, an annual average of 138 publications using the term *chronic subdural hematoma* were indexed in PubMed (18), making this a widely accepted and described entity. The inability of the ICD classification to capture mSDH within the larger and heterogeneous SDH spectrum is a significant problem. From an administrative standpoint, it can lead to inaccuracy in hospital patient record and identification of disease burden in a given population. From a research standpoint, it hinders retrospective identification of *mSDH*, forcing researchers to manually review each case of SDH to confirm chronicity. This adds complexity, time and cost to retrospective projects and renders power calculations for prospective studies such as TRACS (9) either extremely fastidious, or unreliable.

## Conclusion

We propose this new SDH classification system to better capture chronicity and etiology – factors felt to impact management and prognosis. Depending on its acceptance and validation by the scientific community, this system could be submitted as an update to the future ICD classification and solve the currents issues we highlighted with ICD-10 as well as the newer ICD-11.

## Data availability statement

The raw data supporting the conclusions of this article will be made available by the authors, without undue reservation.

## Ethics statement

The studies involving humans were approved by Comité d’éthique de le recherche du CIUSSS de l’Estrie-CHUS, FWA00005894 and IRB00003849. The studies were conducted in accordance with the local legislation and institutional requirements. The ethics committee/institutional review board waived the requirement of written informed consent for participation from the participants or the participants’ legal guardians/next of kin because this is a retrospective study of radiological data.

## Author contributions

A-ML and CT were responsible for the data collection and data analysis. A-ML wrote the first draft of the manuscript. DM and CI-M were involved in the project conception, critical review, and manuscript revision and finalization. All authors contributed to the article and approved the submitted version.

## Abbreviations

SDH: subdural hematoma
cSDH: chronic subdural hematoma
aSDH: acute subdural hematoma
mSDH: membrane-associated subdural hematoma
taSDH: traumatic acute subdural hematoma
ntaSDH: non-traumatic acute subdural hematoma
a/cSDH: acute on chronic subdural hematoma
sSDH: subacute subdural hematoma
